# Clinical benefits of inhaled ciclesonide for hospitalized patients with COVID-19 infection: a retrospective study

**DOI:** 10.1186/s12890-022-02168-8

**Published:** 2022-09-28

**Authors:** Kuan-Chih Kuo, Chao-Hsien Chen, Chieh-Jen Wang, Jou-Chun Wu, Hsin-Pei Chung, Yen-Ting Chen, Yen-Hsiang Tang, Wen-Kuei Chang, Chang-Yi Lin, Chien-Liang Wu

**Affiliations:** 1 Division of Pulmonary and Critical Care Medicine, Department of Internal Medicine, MacKay Memorial Hospital, No. 45, Minsheng Rd., Tamshui District, New Taipei City, 25160 Taiwan; 2 Department of Medicine, MacKay Medical College, New Taipei City, Taiwan; 3 Department of Critical Care Medicine, MacKay Memorial Hospital, Taipei, Taiwan

**Keywords:** Ciclesonide, COVID-19, Inhaled corticosteroid, Mortality, SARS-CoV2

## Abstract

**Background:**

The successful management of patients infected with coronavirus disease 2019 (COVID-19) with inhaled ciclesonide has been reported, however few studies have investigated its application among hospitalized patients.

**Methods:**

This retrospective cohort study enrolled all adult patients admitted to our hospital with confirmed COVID-19 infection from May to June 2021. Critical patients who received mechanical ventilation within 24 h after admission and those who started ciclesonide more than 14 days after symptom onset were excluded. The in-hospital mortality rate was compared between those who did and did not receive inhaled ciclesonide.

**Results:**

A total of 269 patients were enrolled, of whom 184 received inhaled ciclesonide and 85 did not. The use of ciclesonide was associated with lower in-hospital mortality (7.6% vs. 23.5%, *p* = 0.0003) and a trend of shorter hospital stay (12.0 (10.0–18.0) days vs. 13.0 (10.0–25.3) days, *p* = 0.0577). In subgroup analysis, the use of inhaled ciclesonide significantly reduced mortality in the patients with severe COVID-19 infection (6.8% vs. 50.0%, *p* < 0.0001) and in those with a high risk of mortality (16.4% vs. 43.2%, *p* = 0.0037). The use of inhaled ciclesonide also reduced the likelihood of receiving mechanical ventilation in the patients with severe COVID-19 infection. After multivariate analysis, inhaled ciclesonide remained positively correlated with a lower risk of in-hospital mortality (odds ratio: 0.2724, 95% confidence interval: 0.087–0.8763, *p* = 0.0291).

**Conclusions:**

The use of inhaled ciclesonide in hospitalized patients with COVID-19 infection can reduce in-hospital mortality. Further randomized studies in patients with moderate to severe COVID-19 infection are urgently needed.

**Supplementary Information:**

The online version contains supplementary material available at 10.1186/s12890-022-02168-8.

## Introduction

Coronavirus disease 2019 (COVID-19) has infected 404 million people and caused 5 million deaths worldwide [1]. Several treatment options have been introduced, including systemic corticosteroids [2–4], remdesivir [5], tocilizumab [3, 6], enoxaparin [7], and traditional Chinese medicine formula NRICM101 [8]. However, the effectiveness of these treatments is still under debate.

In the early months of the pandemic, Beurnier et al. [9] reported a lower prevalence of asthma patients hospitalized with COVID-19 compared to the general population. There are several possible explanations for this finding. First, patients with asthma have been reported to have lower expressions of angiotensin-converting enzyme 2 (ACE2), the putative viral entry receptor for severe acute respiratory syndrome coronavirus 2 (SARS-CoV-2) [10]. Second, chronic inflammation in asthmatic lungs caused by repeated epithelial insults may lead to a degree of immune tolerance, thereby restricting the development of the excessive inflammatory response in COVID-19 [11–13]. Third, it may be related to a possible protective effect of inhaled corticosteroids (ICS) [9, 14–16].

Anti-inflammatory medications, and especially corticosteroids [3, 4], have become popular in managing patients with severe COVID-19 infection since the RECOVERY trial [17]. However, excess anti-inflammation may be detrimental for patients with milder disease [17]. Compared with systemic corticosteroids, ICS have milder systemic effects [18], and have been shown to be effective in shortening the time to recovery among patients with mild COVID-19 infection [19, 20]. Moreover, some studies have reported that corticosteroids may have anti-viral effects [21–24], and reduce the expressions of ACE-2 and TMPRSS2 [24]. The successful management of patients with COVID-19 infection with inhaled ciclesonide has been reported [25–27], however results from larger patient groups have been controversial or even suggested that ICS may be harmful [28, 29]. Although growing evidence supports the potential role of ICS in the treatment of patients with mild COVID-19 infection and those who do not require hospitalization [19, 20, 30–35], the use of ICS in hospitalized patients remains controversial [36]. In 2020, we once successfully treated a patient with severe COVID-19 infection using inhaled ciclesonide [37]. Considering the limited therapeutic options during the pandemic, our institution then included it as a possible treatment for COVID-19 infection. In this study, we retrospectively analyzed hospitalized COVID-19 patients during the first wave of the pandemic (2021) in Taiwan and compared the effect of inhaled ciclesonide between those who did and did not receive treatment.

## Materials and methods

### Study design and patient selection

This study was a single-center, retrospective analysis. All patients admitted to Mackay Memorial Hospital with a diagnosis of COVID-19 from May 1st, 2021, to June 30th, 2021, were enrolled. The patients were eligible for inclusion if they: (1) were ≥ 18 years of age, and (2) had a positive COVID-19 reverse transcription-polymerase chain reaction (RT-PCR) at the emergency department. Patients who were (1) previously treated, (2) transferred to/from another hospital, (3) had delayed ciclesonide treatment (time from symptoms to first ciclesonide treatment > 14 days), and (4) received mechanical ventilation support on admission or within 24 h after admission, were excluded from the analysis. Their medical records were reviewed, and demographic characteristics, co-morbidities, treatment received, and outcomes during hospitalization were collected. Severe COVID-19 infection was defined as pulse oximetry < 94% under ambient air or requiring supplemental oxygen at admission [38]. Patients at high risk of mortality were defined according to Shang’s COVID-19 scoring system (CSS) as > 2 points on the first day of admission [39]. This study was approved by the Institutional Review Board of MacKay Memorial Hospital (approval no. 21MMHIS330e) and the need for written informed consent was waived.

### Treatment protocol at our hospital

After admission, all patients remained asymptomatic or had mild symptoms but did not require oxygen supply, only symptoms support measurements were applied. Pulse oximetry (SpO2) was monitored every 8 h in all patients. The patients with an FiO_2_ ≥ 0.4 were treated with oral dexamethasone 6 mg/day and remdesivir if within 5 days of symptom onset. Inhaled ciclesonide three puffs (480 mcg) every 8 h was an option if the patients needed oxygen supply. Prophylactic enoxaparin 40 mg SC daily was given if the D-dimer level was > 1000 ng/ml or there was a high risk of developing thromboembolism. Traditional Chinese medicine NRICM101 was also an option.

If the patient’s condition continued to deteriorate and intubation with mechanical ventilator support was considered to be necessary, tocilizumab, an anti-IL-6 receptor antibody, was given (8 mg/kg of ideal body weight, maximum 800 mg) once. A prophylactic dose of enoxaparin was also given if not previously prescribed (Additional file: 1).

### Outcome measurements

The primary outcome was in-hospital mortality. The secondary outcomes included the use of supplemental oxygen, mechanical ventilation, duration of fever, and hospital stay. The time to mortality after admission was calculated, and the patients were followed until they died or were discharged.

Subgroup analyses by disease severity, including severe COVID-19 infection or risk according to Shang’s CSS score, were also performed. The risks of supplemental oxygen, mechanical ventilation, duration of fever, and hospital stay were also analyzed.

### Statistical analysis

Categorical variables were reported as number (percentage). The chi-squared test or Fisher’s exact test was used to compare the frequencies of categorical variables, when appropriate. Continuous variables with normal distribution were reported as mean ± standard deviation (SD), and non-normally distributed variables were reported as median (interquartile range [IQR]). The Shapiro–Wilk test was used to examine the normality of distribution of continuous variables. Two continuous normally distributed variables were compared using independent samples t-tests. The Mann–Whitney U test was used to compare two groups of non-normally distributed variables. For time-to-event analysis, the log-rank test and Cox’s proportional hazards model were used to compare differences and effect size in the probability.

Variables with p < 0.05 in univariate logistic regression analysis were considered as confounders, and they were used in multivariable logistic regression analysis. All p values were two-sided, and a value < 0.05 was considered to be statistically significant. All analyses were performed using MedCalc version 20.014 for Windows (MedCalc Software Ltd, Ostend, Belgium).

## Results

**Fig. 1:**
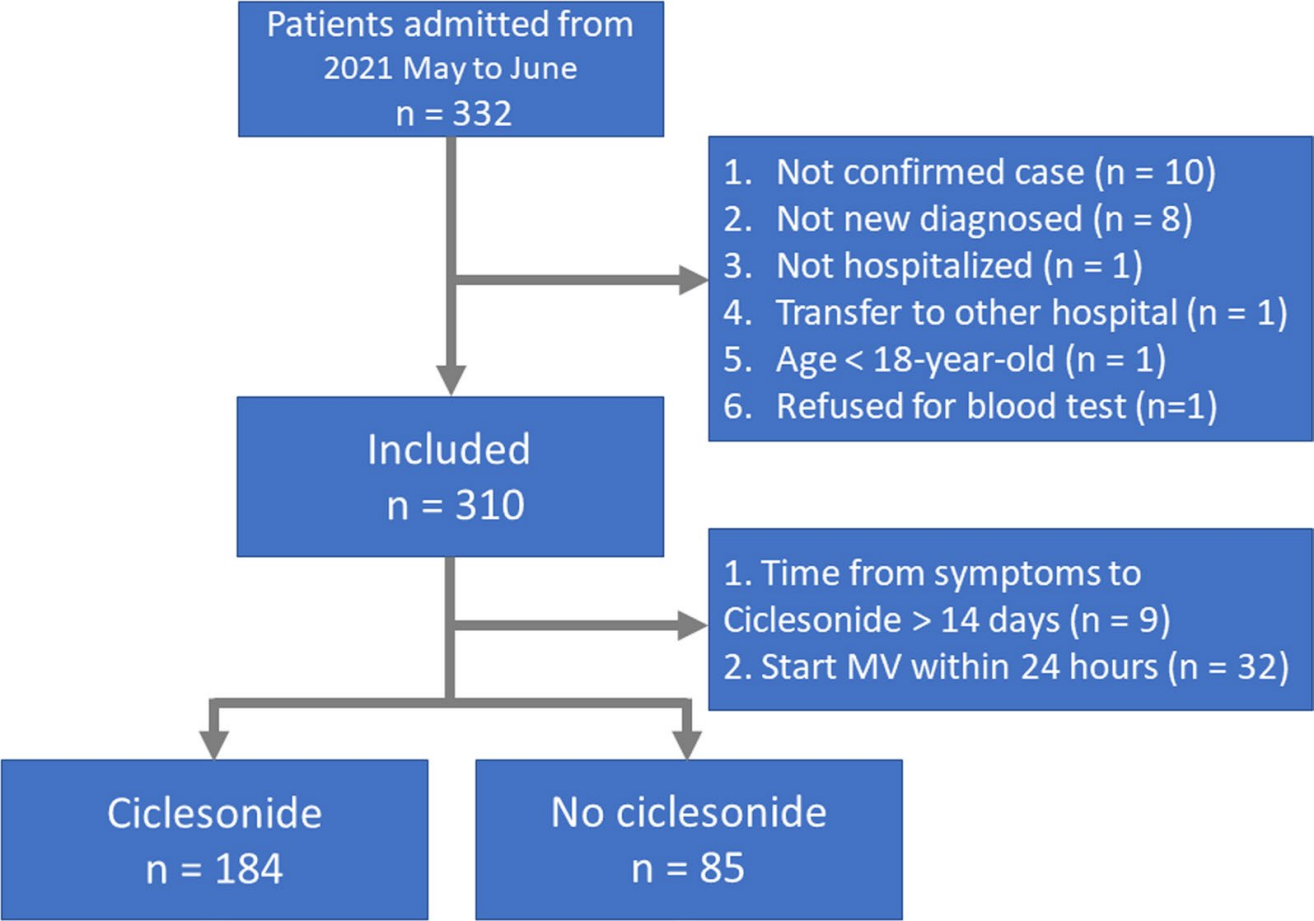
Flowchart of patient inclusion in the study

In total, 332 patients were diagnosed with COVID-19 during the study period, of whom 63 were excluded (see Fig. 1 for details). Finally, 269 patients were enrolled for analysis, of whom 184 received inhaled ciclesonide and 85 did not.

The median ages were 62.0 (55.0–71.0) and 62.0 (50.8–74.5) years in the ciclesonide and non-ciclesonide groups, respectively (Table 1). There was no difference in gender distribution. Compared to the non-ciclesonide group, the ciclesonide group had less heart failure (1.1% vs. 12.9%, *p* < 0.0001) and chronic kidney disease (3.3% vs. 20.0%, *p* < 0.0001). Regarding baseline disease severity, the ciclesonide group were associated with more severe COVID-19 infection (64.1% vs. 40.0%, *p* = 0.0002), but were less likely to be categorized as high-risk using Shang’s CSS score (34.1% vs. 47.4%, *p* = 0.0431). The ciclesonide group received more oral/intravenous corticosteroids (90.8% vs. 55.3%, *p* ≤ 0.0001), remdesivir (44.0% vs. 28.2%, *p* = 0.0138) and NRICM101 (31.0% vs. 7.1%, *p* < 0.0001) during hospitalization.

**Table 1:** Baseline characteristics, treatment and hospital outcomes of COVID-19 patients treated with and without inhaled ciclesonide

	Ciclesonide group (n = 184)	No ciclesonide group (n = 85)	*p* value
Age, years	62.0 (55.0–71.0)	62.0 (50.8–74.5)	0.9261
Sex (Male/female)	99/85 (53.8%/46.2%)	43/42 (50.6%/49.4%)	0.6239
*Co-morbidities*			
Hypertension	73 (39.7%)	34 (40.0%)	0.9596
Diabetes	53 (28.8%)	25 (29.4%)	0.9188
Cardiovascular disease	13 (7.1%)	7 (8.2%)	0.7343
Heart failure	2 (1.1%)	11 (12.9%)	< 0.0001
Chronic obstructive pulmonary disease	8 (4.3%)	4 (4.7%)	> 0.9999
Organ transplantation	0 (0.0%)	0 (0.0%)	NA
Chronic kidney disease	6 (3.3%)	17 (20.0%)	< 0.0001
Malignancy	10 (5.4%)	6 (7.1%)	0.6013
Duration from symptoms to admission, days	5.0 (2.0–8.0)	3.0 (1.0–7.0)	0.0030
Fever	126 (68.5%)	59 (70.2%)	0.7730
Shang’s CSS score^a^	2.0 (1.0–3.0)	2.0 (1.0–4.0)	0.1723
High risk Shang’s CSS score^a^	61 (34.1%)	37 (47.4%)	0.0431
Severe COVID-19 infection	118 (64.1%)	34 (40.0%)	0.0002
*Treatment*			
Systemic corticosteroids	167 (90.8%)	47 (55.3%)	< 0.0001
Remdesivir	81 (44.0%)	24 (28.2%)	0.0138
Tocilizumab	54 (29.3%)	16 (18.8%)	0.0679
Enoxaparin	62 (33.7%)	25 (29.4%)	0.4858
Traditional Chinese medicine formula NRICM101	57 (31.0%)	6 (7.1%)	< 0.0001
*Outcomes*			
In-hospital mortality	14 (7.6%)	20 (23.5%)	0.0003
Supplemental oxygen during hospitalization	156 (84.8%)	51 (60.0%)	< 0.0001
Mechanical ventilation	23 (12.5%)	12 (14.1%)	0.7144
Duration of fever, days	2.0 (1.0–3.0)	2.0 (1.0–4.0)	0.1381
Duration of hospital stay, days	12.0 (10.0–18.0)	13.0 (10.0–25.3)	0.0577

*COVID-19* coronavirus disease 2019; *NA* not applicable; *Shang’s CSS* Shang’s COVID-19 scoring system

^a^There were 179 versus 78 patients available to calculated Shang’s CSS risk score

**Fig. 2:**
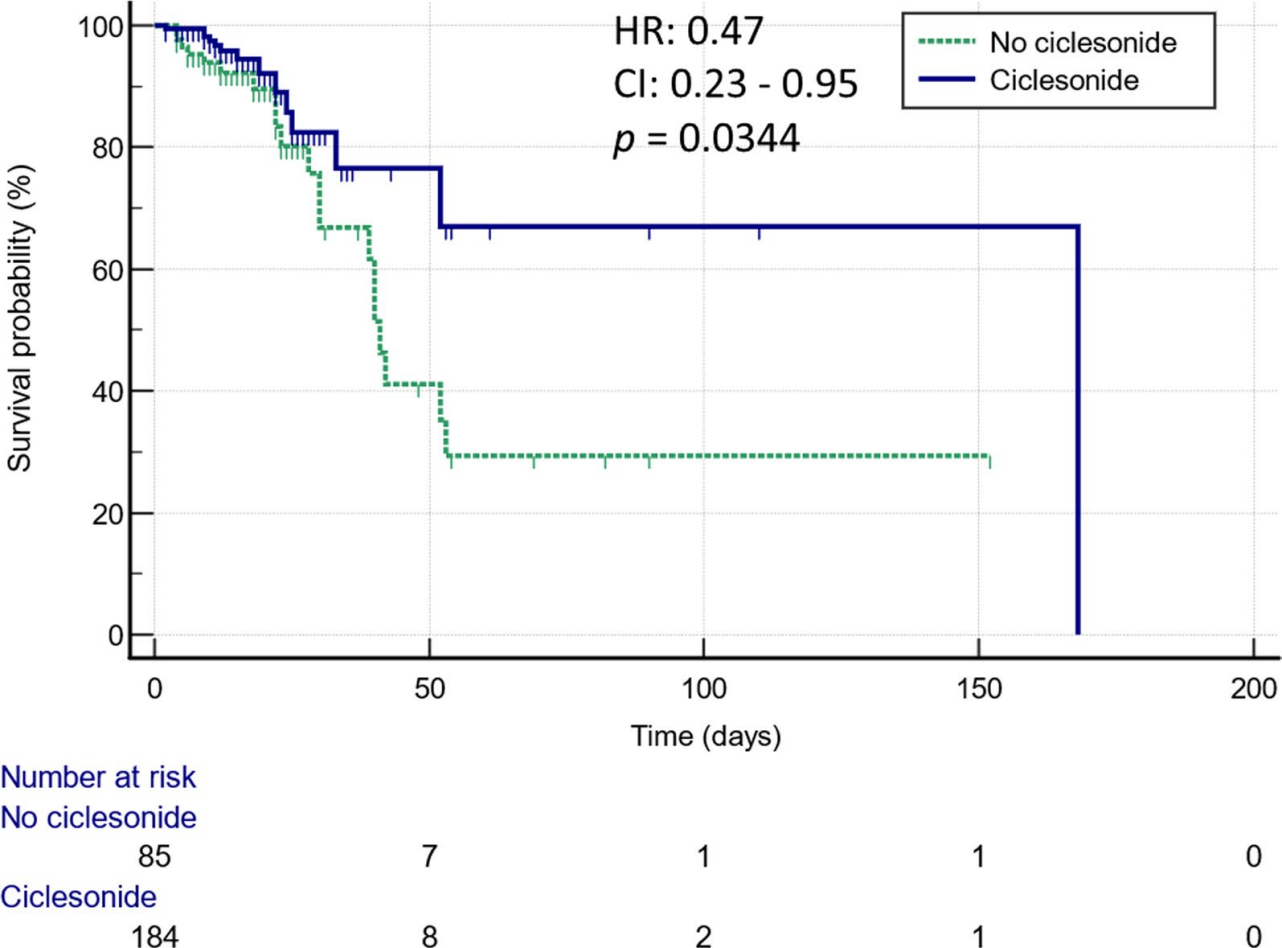
Kaplan–Meier curves of the cumulative probability of survival after admission in patients with COVID-19 infection treated with or without inhaled ciclesonide. CI: confidence interval, HR: hazard ratio

Regarding the primary outcome, the use of inhaled ciclesonide was associated with lower in-hospital mortality (7.6% vs. 23.5%, *p* = 0.0003) and more frequent use of oxygen supplementation during hospitalization (84.8% vs. 60.0%, *p* < 0.0001). There was no significant difference in the duration of fever between the two group (2.0 (1.0–3.0) versus 2.0 (1.0–4.0) days, *p* = 0.1381). In the time-to-mortality analysis, the use of ciclesonide was associated with a significantly lower risk of mortality (hazard ratio: 0.47, 95% confidence interval [CI]: 0.23–0.95, *p* = 0.0344) (Fig. 2).

The use of inhaled ciclesonide, age, hypertension, diabetes, heart failure, chronic kidney disease, oxygen therapy at admission, high risk Shang’s CSS score, treatment with systemic corticosteroids, and tocilizumab were related to in-hospital mortality in univariate analysis (Table 2). These factors were then entered into multivariate analysis, which showed that inhaled ciclesonide was associated with a lower risk of in-hospital mortality (odds ratio: 0.2724, 95% CI: 0.087–0.8763, *p* = 0.0291) (Table 3).

**Table 2:** Univariate logistic regression analysis for in-hospital mortality

	Odds ratio	95% confidence interval	*p* value
Inhaled ciclesonide	0.2676	0.1276–0.5612	0.0005
Age	1.0789	1.0455–1.1133	< 0.0001
Sex, male	1.0071	0.4899–2.0700	0.9847
Hypertension	3.2353	1.5251–6.8631	0.0022
Diabetes	2.4803	1.1914–5.1637	0.0152
Cardiovascular disease	2.5287	0.8556–7.4738	0.0934
Heart failure	32.2222	8.2954–125.1619	< 0.0001
Chronic obstructive pulmonary disease	1.4063	0.2947–6.7103	0.6689
Chronic kidney disease	11.1074	4.3914–28.0950	< 0.0001
Malignancy	1.6526	0.4457–6.1276	0.4524
Severe COVID-19 infection	2.3622	1.0572–5.2780	0.0361
High risk Shang’s CSS score	13.9931	4.7086–41.5847	< 0.0001
Systemic corticosteroids	4.6593	1.0810–20.0825	0.039
Remdesivir	1.6705	0.8112–3.4398	0.1638
Tocilizumab	4.5699	2.1703–9.6229	0.0001
Enoxaparin	1.5534	0.7436–3.2454	0.2413
Traditional Chinese medicine formula NRICM101	0.8287	0.3424–2.0056	0.6769

COVID-19: coronavirus disease 2019; Shang’s CSS: Shang’s COVID-19 scoring system

**Table 3:** Multivariate logistic regression model for in-hospital mortality

	Odds ratio	95% confidence interval	*p* value
Inhaled ciclesonide	0.2724	0.0847–0.8763	0.0291
Age	1.0504	1.0004–1.1030	0.0481
Hypertension	0.8023	0.2547–2.5272	0.7067
Diabetes	1.3944	0.4669–4.1644	0.5514
Heart failure	9.7668	1.8968–50.2903	0.0064
Chronic kidney disease	1.5271	0.3673–6.3503	0.5604
Severe COVID-19 infection	0.7866	0.2385–2.5938	0.6933
High risk Shang’s CSS score	4.4695	1.1094–18.0056	0.0352
Systemic corticosteroids	2.1265	0.2778–16.2771	0.4675
Tocilizumab	8.0628	2.5079–25.9221	0.0005

*COVID-19* coronavirus disease 2019; *Shang’s CSS* Shang’s COVID-19 scoring system

In subgroup analysis (Table 4), the use of inhaled ciclesonide was associated with a significant reduction in mortality in the patients with severe COVID-19 infection (6.8% vs. 50.0%, *p* < 0.0001) and those with a high Shang’s CSS score (16.4% vs. 43.2%, *p* = 0.0037). Ciclesonide was also found to reduce the risk of mechanical ventilation use among the patients with severe COVID-19 infection (15.3% vs. 32.4%, *p* = 0.0259). There were more supplemental oxygen orders during hospitalization for the patients who received ciclesonide with non-severe COVID-19 infection (57.6% vs. 33.3%, *p* = 0.0095).

**Table 4:** Subgroup analysis according to disease severity and hospital outcomes between COVID-19 patients treated with and without inhaled ciclesonide

	Severe COVID-19 infection			Not severe COVID-19 infection		
	Ciclesonide (n = 118)	No ciclesonide (n = 34)	p value	Ciclesonide (n = 66)	No ciclesonide (n = 51)	p value
Initiated supplemental oxygen after admission	Not applicable			38 (57.6%)	17 (33.3%)	0.0095
Mechanical ventilation	18 (15.3%)	11 (32.4%)	0.0259	5 (7.6%)	1 (2.0%)	0.2301
In-hospital mortality	8 (6.8%)	17 (50.0%)	< 0.0001	6 (9.1%)	3 (5.9%)	0.7296

	High Shang’s CSS risk			Low Shang’s CSS risk		
	Ciclesonide (n = 61)	No ciclesonide (n = 37)	p value	Ciclesonide (n = 118)	No ciclesonide (n = 41)	p value
Initiated supplemental oxygen after admission	9 (14.8%)	8(21.6%)	0.3865	27 (22.9%)	9 (22.0%)	0.9027
Mechanical ventilation	9 (14.8%)	8 (21.6%)	0.3865	14 (11.9%)	4 (9.8%)	> 0.9999
In-hospital mortality	10 (16.4%)	16 (43.2%)	0.0037	2 (1.7%)	2 (4.9%)	0.2738

*COVID-19* coronavirus disease 2019; *Shang’s CSS* Shang’s COVID-19 scoring system

## Discussion

Our results showed that the use of inhaled ciclesonide in hospitalized patients with COVID-19 infection, especially those with severe COVID-19 infection, was associated with lower in-hospital mortality. Inhaled ciclesonide also reduced the likelihood of mechanical ventilation in the patients with severe disease during hospitalization. However, inhaled ciclesonide did not shorten the hospital stay or time to symptom relief in these patients. In the patients with milder disease, the use of inhaled ciclesonide increased the risk of receiving oxygen therapy. But did not increase the risk of receiving mechanical ventilation support or mortality. Overall, inhaled ciclesonide was an effective treatment option for hospitalized patients with COVID-19 infection.

The rationale for the use of corticosteroids in patients with sepsis is to downregulate pro-inflammatory responses [40], however their use for severe sepsis or viral infection is controversial [41–43]. The ADRENAL trial reported no difference in primary outcome (90-day mortality) between hydrocortisone and placebo groups, but a shorter median time to resolution of shock, discharge from the ICU, and cessation of mechanical ventilation [44]. The DEXA-ARDS trial suggested that the early use of dexamethasone could reduce pulmonary and systemic inflammation in moderate to severe ARDS, thereby reducing overall mortality [45]. However, suppressing inflammatory reactions was shown to potentially increase the risk of developing secondary bacterial pneumonia in patients with influenza pneumonia [43]. In addition, alterations in immune reactions caused by corticosteroids in patients with SARS-associated coronavirus infection have been suggested to lead to prolonged viremia and delayed viral clearance, ultimately increasing the risk of mortality [42]. In severe COVID-19, as with other types of viral pneumonia, the host immune response is thought to play a key role in the pathophysiology of organ failure. Signs of inflammatory organ injury with markedly elevated levels of inflammatory markers in severe COVID-19 patients have prompted the use of anti-inflammatory agents, including corticosteroids. However, the value of corticosteroids was uncertain [46, 47] until the RECOVERY trial, in which the use of dexamethasone 6 mg/day resulted in lower 28-day mortality among patients receiving either mechanical ventilation or oxygen supplementation alone [17].

Concerns over the side effects of systemic corticosteroids still remain [48]. Unlike systemic corticosteroids, ICS treatment has minimal systemic effects, and the impact on COVID-19 infection in patients with asthma is still under debate [9, 28]. ICS treatment has been associated with decreased gene expressions of proteins ACE2 and TMPRSS2 in type 2 alveolar cells, thereby reducing coronavirus replication, including SARS-CoV-2 [28]. ACE2 and TMPRSS2 are associated with viral cell entry, and are involved in binding of the spike protein and the beginning of viral infection [28, 49]. The STOIC trial evaluated the efficacy of inhaled budesonide in community-dwelling individuals of all ages with early (symptom onset < 7 days) COVID-19, including 146 individuals with mild symptoms [19, 38]. The results showed that the budesonide group had a lower COVID-19-related urgent care visit rate, shorter clinical recovery time, and fewer days with a fever in the first 14 days than the usual care group. The trial concluded that budesonide was safe, with only 7% of the participants reporting self-limiting adverse events. However, the benefits on mortality of inhaled budesonide was not reported.

The PRINCIPLE trial enrolled 4,700 non-hospitalized participants > 65 years of age with suspected COVID-19, and randomly assigned them to receive budesonide (n = 1073), usual care alone (n = 1988), or other treatments (n = 1639) [20]. The budesonide group had shorter time to first recovery, however the hospital admission and mortality rates between the budesonide and usual care groups did not reach statistical significance. Two participants in the budesonide group and four in the usual care group had serious adverse events. In Al Sulaiman et al.’s [36] study, the 30-day mortality rate was significantly lower in 65 patients who received ICS during ICU stay (HR: 0.53, 95% CI: 0.31–0.93, *p* = 0.03). However, in-hospital mortality, ventilator-free days, ICU and hospital length of stay were not statistically significant between the ICS and usual care groups. Compared with these studies, we enrolled hospitalized patients with mainly moderate to severe COVID-19. Their average age was 62 years, and most had one or more comorbidities. Due to the nature of this non-randomized, retrospective study, inhaled ciclesonide was not prescribed for all patients according to protocol but at the physician’s judgement. In addition, the distribution of comorbidities was not even between groups. However, inhaled ciclesonide was still shown to reduce the in-hospital mortality rate after multivariate logistic regression analysis. To the best of our knowledge, this is the first study to report that inhaled ciclesonide could reduce mortality in hospitalized COVID-19 patients. In the patients with severe COVID-19 at admission, using inhaled ciclesonide reduced the likelihood of receiving invasive mechanical ventilation during hospitalization, and this may explain its effect on the reduction of in-hospital mortality. However, further studies are needed to verify this speculation.

In contrast to the STOIC trial, we found no significant difference in symptom relief rate between the groups [19]. This may be due to differences in the definition of symptoms, as we only studied afebrile rate, which was not different between groups. A possible explanation is that our patients were more severe than those in the STOIC trial. In addition, the hospital stay in our study was not shorter than that in the PRINCIPLE trial [20]. A possible reason may be the discharge criteria in Taiwan, including negative RT-PCR tests for two consecutive days [50]. The administrative regulations in Taiwan require RT-PCR tests at fixed times after admission, which may have prolonged admission. Moreover, the natural course of SARS-COV-2 shedding is unknown [51], so it is not possible to arrange RT-PCR to confirm the readiness for discharge. In the patients with milder severity at admission (no oxygen supplementation), the use of inhaled ciclesonide increased the risk of deterioration (requiring oxygen supplementation); but the likelihood of receiving invasive mechanical ventilation and in-hospital mortality were not different compared to those who did not use inhaled ciclesonide. Treatment with ICS may be harmful rather than beneficial in patients with mild disease severity [29]. This finding is similar with the RECOVERY trial [17], in which non-severe patients did not benefit and in some cases were harmed by steroid treatment. Shang’s CSS has been reported to effectively predict mortality [39]. In our study, inhaled ciclesonide benefitted patients with a high Shang’s CSS score in mortality but not in the likelihood of receiving mechanical ventilation. In contrast, there was no significant impact among the patients with a low Shang’s CSS score. Although the side effects of ICS are thought to be mild [19, 20, 28], our results suggest that the severity before initiating ICS may be important. Further investigations are needed to clarify this issue.

Not all ICS are the same with regards to anti-inflammatory potency or inhibition of virus replication [48]. Matsuyama et al. [23] found that inhaled ciclesonide, compared with other corticosteroids, blocked coronavirus replication in a cell culture line with low cytotoxicity. A randomized phase 2 trial conducted in Korea also found that inhaled ciclesonide shortened SARS-CoV-2 viral shedding duration and may inhibit progression to respiratory failure in mild to moderate COVID-19 [35]. During the early stage of the pandemic, some case reports from Japan described that COVID-19 pneumonia could be improved after inhaled ciclesonide therapy [25–27], which is similar to our previous report [37]. However, the results from subsequent randomized control studies regarding inhaled ciclesonide are conflicting. In Clemency et al.’s [33] study of 400 symptomatic COVID-19 outpatients, the median time to alleviation of all COVID-19-related symptoms was not different between inhaled ciclesonide and placebo arms, although the inhaled ciclesonide group had fewer COVID-19-related emergency department visits and hospital admissions. However, they enrolled relatively mild and young (average 43.3 years) patients. The CONTAIN trial [34] was a phase II randomized controlled trial which compared intranasal and inhaled ciclesonide with placebo in 203 outpatients (average age 35 years), and reported no significant difference in the resolution of symptoms by day 7 between the ciclesonide and control groups. This suggests that inhaled ciclesonide may not be beneficial for healthy young COVID-19 patients [34]. In summary, our study is the first to report that inhaled ciclesonide could reduce in-hospital mortality among severe hospitalized COVID-19 patients.

The average age of our patients was 62 years, which is higher than in other similar trials [19, 20, 33, 34]. However, only our study was performed in hospitalized, severe COVID-19 patients. Al Sulaiman et al. [36] recruited a similar age group (61.4 ± 14.7 years) of patients in an ICU who mainly used inhaled budesonide, but only 30 days mortality was significant. Differences in the steroids used, dosage and study outcomes between the studies make direct comparisons impractical. We suggest that ICS, not necessarily only ciclesonide, could play a role in managing moderate to severe COVID-19 patients, however further well-designed studies are needed to clarify this issue.

This study has several limitations. First, it is a retrospective observational study conducted at a single center. The choice to prescribe inhaled ciclesonide was made by the clinician in charge. Although we recommend the timing of use as SpO2 < 94% on room air at sea level (defined as severe COVID-19), this differed among our colleagues due to concerns of side effects. Selection bias was also present, as reflected by the unbalanced comorbidity distribution between groups. However, multivariate regression analysis still suggested a significant difference in reducing mortality with inhaled ciclesonide. Moreover, treatment recommendations may explain the greater use of supplemental oxygen after admission in the ciclesonide group. More patients used oxygen in the ciclesonide group because the initiation of ciclesonide was suggested if the patients had SpO2 < 94%. We could not distinguish between the use of oxygen as a cause of disease or as a result of ciclesonide treatment. Second, during the study period, people in Taiwan were seldom fully vaccinated [52], and the Alpha variant was predominant. The applicability of our results to the omicron variant or vaccinated populations may need further study. Third, during the first wave of the pandemic in Taiwan, oral dexamethasone 6 g/day was the standard management for severe COVID-19 [17]. Other anti-inflammatory agents, such as anti-IL-6 could also be considered, and it was not necessary to use ICS. Univariate and multivariate logistic regression analyses revealed that inhaled ciclesonide, but not systemic corticosteroids was associated with lower in-hospital mortality. It is therefore reasonable that the balance between inflammation and anti-inflammation is critical for septic patients. Fourth, we did not evaluate all scoring systems to distinguish the severity of COVID-19. However, two different classification systems both showed that inhaled ciclesonide reduced mortality in the severe group [38, 39]. Finally, due to the retrospective nature of the study, some biomarkers were not checked, and some data were missing on medical records. Therefore, we could not analyze the predictive value of the biomarkers [53], smoking status [54], or when the symptoms subsided.

## Conclusion

The use of inhaled ciclesonide in hospitalized patients with COVID-19 infection reduced in-hospital mortality and the likelihood of receiving invasive mechanical ventilation in those with severe COVID-19 infection. Further well-designed randomized control trials are indicated to validate our findings.

## Supplementary Information

**Additional file 1**: **1** Treatment protocol for COVID patients at MacKay Memorial Hospital (2021). **2** Admission order set (2021).

## Data Availability

Not applicable.

## References

[1] World Health Organization: WHO coronavirus (COVID-19) dashboard. 2022, Available at https://covid19.who.int/. Accessed 12 Feb 2022.

[2] Sterne JAC, Murthy S, Diaz JV, Slutsky AS, Villar J, Angus DC, Annane D, Azevedo LCP, Berwanger O, Cavalcanti AB (2020). Association between administration of systemic corticosteroids and mortality among critically ill patients with COVID-19: a meta-analysis. JAMA.

[3] Gutiérrez-Abejón E, Herrera-Gómez F, Pedrosa-Naudín MA, Tamayo E, Álvarez FJ (2022). Hospitalized COVID-19 patients with severe acute respiratory syndrome: a population-based registry analysis to assess clinical findings, pharmacological treatment and survival. Medicina (Kaunas).

[4] Salton F, Confalonieri P, Meduri GU, Santus P, Harari S, Scala R, Lanini S, Vertui V, Oggionni T, Caminati A (2020). Prolonged low-dose methylprednisolone in patients with severe COVID-19 pneumonia. Open Forum Infect Dis.

[5] Lai CC, Chen CH, Wang CY, Chen KH, Wang YH, Hsueh PR (2021). Clinical efficacy and safety of remdesivir in patients with COVID-19: a systematic review and network meta-analysis of randomized controlled trials. J Antimicrob Chemother.

[6] Lin WT, Hung SH, Lai CC, Wang CY, Chen CH (2021). The effect of tocilizumab on COVID-19 patient mortality: a systematic review and meta-analysis of randomized controlled trials. Int Immunopharmacol.

[7] Albani F, Sepe L, Fusina F, Prezioso C, Baronio M, Caminiti F, Di Maio A, Faggian B, Franceschetti ME, Massari M (2020). Thromboprophylaxis with enoxaparin is associated with a lower death rate in patients hospitalized with SARS-CoV-2 infection. A cohort study. EClinicalMedicine.

[8] Tsai KC, Huang YC, Liaw CC, Tsai CI, Chiou CT, Lin CJ, Wei WC, Lin SJ, Tseng YH, Yeh KM (2021). A traditional Chinese medicine formula NRICM101 to target COVID-19 through multiple pathways: a bedside-to-bench study. Biomed Pharmacother.

[9] Beurnier A, Jutant EM, Jevnikar M, Boucly A, Pichon J, Preda M, Frank M, Laurent J, Richard C, Monnet X et al. Characteristics and outcomes of asthmatic patients with COVID-19 pneumonia who require hospitalisation. Eur Respir J. 2020;56(5):2001875. 10.1183/13993003.01875-2020. 10.1183/13993003.01875-2020 PMC7397950 32732333

[10] Jackson DJ, Busse WW, Bacharier LB, Kattan M, O’Connor GT, Wood RA, Visness CM, Durham SR, Larson D, Esnault S (2020). Association of respiratory allergy, asthma, and expression of the SARS-CoV-2 receptor ACE2. J Allergy Clin Immunol.

[11] Farne H, Singanayagam A: Why asthma might surprisingly protect against poor outcomes in COVID-19. Eur Respir J. 2020;56(6):2003045. 10.1183/13993003.03045-2020. 10.1183/13993003.03045-2020 PMC7651838 33154034

[12] Salton F, Confalonieri P, Campisciano G, Cifaldi R, Rizzardi C, Generali D, Pozzan R, Tavano S, Bozzi C, Lapadula G (2022). Cytokine profiles as potential prognostic and therapeutic markers in SARS-CoV-2-induced ARDS. J Clin Med.

[13] Luo XH, Zhu Y, Mao J, Du RC (2021). T cell immunobiology and cytokine storm of COVID-19. Scand J Immunol.

[14] Bloom CI, Drake TM, Docherty AB, Lipworth BJ, Johnston SL, Nguyen-Van-Tam JS, Carson G, Dunning J, Harrison EM, Baillie JK (2021). Risk of adverse outcomes in patients with underlying respiratory conditions admitted to hospital with COVID-19: a national, multicentre prospective cohort study using the ISARIC WHO clinical characterisation protocol UK. Lancet Respir Med.

[15] Finney LJ, Glanville N, Farne H, Aniscenko J, Fenwick P, Kemp SV, Trujillo-Torralbo MB, Loo SL, Calderazzo MA, Wedzicha JA (2021). Inhaled corticosteroids downregulate the SARS-CoV-2 receptor ACE2 in COPD through suppression of type I interferon. J Allergy Clin Immunol.

[16] Peters MC, Sajuthi S, Deford P, Christenson S, Rios CL, Montgomery MT, Woodruff PG, Mauger DT, Erzurum SC, Johansson MW (2020). COVID-19-related genes in sputum cells in asthma. Relationship to demographic features and corticosteroids. Am J Respir Crit Care Med.

[17] Horby P, Lim WS, Emberson JR, Mafham M, Bell JL, Linsell L, Staplin N, Brightling C, Ustianowski A, Elmahi E (2021). Dexamethasone in hospitalized patients with COVID-19. N Engl J Med.

[18] Lipworth BJ (1999). Systemic adverse effects of inhaled corticosteroid therapy: a systematic review and meta-analysis. Arch Intern Med.

[19] Ramakrishnan S, Nicolau DV, Langford B, Mahdi M, Jeffers H, Mwasuku C, Krassowska K, Fox R, Binnian I, Glover V (2021). Inhaled budesonide in the treatment of early COVID-19 (STOIC): a phase 2, open-label, randomised controlled trial. Lancet Respir Med.

[20] Yu LM, Bafadhel M, Dorward J, Hayward G, Saville BR, Gbinigie O, Van Hecke O, Ogburn E, Evans PH, Thomas NPB (2021). Inhaled budesonide for COVID-19 in people at high risk of complications in the community in the UK (PRINCIPLE): a randomised, controlled, open-label, adaptive platform trial. Lancet.

[21] Jeon S, Ko M, Lee J, Choi I, Byun SY, Park S, Shum D, Kim S (2020). Identification of antiviral drug candidates against SARS-CoV-2 from FDA-approved drugs. Antimicrob Agents Chemother.

[22] Yamaya M, Nishimura H, Deng X, Sugawara M, Watanabe O, Nomura K, Shimotai Y, Momma H, Ichinose M, Kawase T (2020). Inhibitory effects of glycopyrronium, formoterol, and budesonide on coronavirus HCoV-229E replication and cytokine production by primary cultures of human nasal and tracheal epithelial cells. Respir Investig.

[23] Matsuyama S, Kawase M, Nao N, Shirato K, Ujike M, Kamitani W, Shimojima M, Fukushi S: The inhaled corticosteroid ciclesonide blocks coronavirus RNA replication by targeting viral NSP15. bioRxiv 2020:2020.2003.2011.987016. 10.1128/JVI.01648-20 PMC7737752 33055254

[24] Gonzalez-Barcala FJ, Nieto-Fontarigo JJ, Mendez-Brea P, Salgado FJ (2022). The polyhedric reality of the interaction between COVID-19, asthma and inhaled corticosteroids. ERJ Open Res.

[25] Nakajima K, Ogawa F, Sakai K, Uchiyama M, Oyama Y, Kato H, Takeuchi I (2020). A case of coronavirus disease 2019 treated with ciclesonide. Mayo Clin Proc.

[26] Tsuchida T, Yamasaki Y, Kunishima H, Sato K, Kanazawa M, Moriuchi A, Morikawa D, Takita T, Naito Y, Fujii S (2020). Treatment of two cases of COVID-19 with ciclesonide resulted in amelioration of pneumonia symptoms. Jpn J Antibiot.

[27] Iwabuchi K, Yoshie K, Kurakami Y, Takahashi K, Kato Y, Morishima T (2020). Therapeutic potential of ciclesonide inahalation for COVID-19 pneumonia: report of three cases. J Infect Chemother Off J Japn Soc Chemother.

[28] Choi JC, Jung SY, Yoon UA, You SH, Kim MS, Baek MS, Jung JW, Kim WY (2020). Inhaled corticosteroids and COVID-19 risk and mortality: a nationwide cohort study. J Clin Med.

[29] Schultze A, Walker AJ, MacKenna B, Morton CE, Bhaskaran K, Brown JP, Rentsch CT, Williamson E, Drysdale H, Croker R (2020). Risk of COVID-19-related death among patients with chronic obstructive pulmonary disease or asthma prescribed inhaled corticosteroids: an observational cohort study using the OpenSAFELY platform. Lancet Respir Med.

[30] Chen CH, Wang CY, Wang YH, Chen CY, Chen KH, Lai CC, Wei YF, Fu PK (2022). The effect of inhaled corticosteroids on the outcomes of patients with COVID-19: A systematic review and meta-analysis of randomized controlled trials. Expert Rev Clin Pharmacol.

[31] Liew F, Openshaw PJM (2022). Inhaled corticosteroids: not just for asthma, but for COVID-19?. Lancet Respir Med.

[32] Alsultan M, Obeid A, Alsamarrai O, Anan MT, Bakr A, Soliman N, Kurdy M, Mosa MH, Saleh Z, Hujij F (2021). Efficacy of colchicine and budesonide in improvement outcomes of patients with coronavirus infection 2019 in Damascus, Syria: a randomized control trial. Interdiscip Perspect Infect Dis.

[33] Clemency BM, Varughese R, Gonzalez-Rojas Y, Morse CG, Phipatanakul W, Koster DJ, Blaiss MS (2022). Efficacy of inhaled ciclesonide for outpatient treatment of adolescents and adults with symptomatic COVID-19: a randomized clinical trial. JAMA Intern Med.

[34] Ezer N, Belga S, Daneman N, Chan A, Smith BM, Daniels SA, Moran K, Besson C, Smyth LY, Bartlett SJ (2021). Inhaled and intranasal ciclesonide for the treatment of covid-19 in adult outpatients: CONTAIN phase II randomised controlled trial. BMJ.

[35] Song JY, Yoon JG, Seo YB, Lee J, Eom JS, Lee JS, Choi WS, Lee EY, Choi YA, Hyun HJ (2021). Ciclesonide inhaler treatment for mild-to-moderate COVID-19: a randomized, open-label, phase 2 trial. J Clin Med.

[36] Al Sulaiman K, Aljuhani O, Al Aamer K, Al Shaya O, Al Shaya A, Alsaeedi AS, Alhubaishi A, Altebainawi AF, Al Harthi A, Albelwi S (2022). The role of inhaled corticosteroids (ICS) in critically ill patients with COVID-19: a multicenter, cohort study. J Intensive Care Med.

[37] Kuo KC, Wang CJ, Chung HP, Lin CY (2022). Clinical application of inhaled ciclesonide and enoxaparin for COVID-19 pneumonia. Int J Gerontol.

[38] National Institutes of Health: **Clinical spectrum of SARS-CoV-2 infection**. *COVID-19 Treatment Guidelines* 2021, Available at https://www.covid19treatmentguidelines.nih.gov/overview/clinical-spectrum/; access date: 2022/02/12.

[39] Shang Y, Liu T, Wei Y, Li J, Shao L, Liu M, Zhang Y, Zhao Z, Xu H, Peng Z (2020). Scoring systems for predicting mortality for severe patients with COVID-19. EClinicalMedicine.

[40] Keh D, Boehnke T, Weber-Cartens S, Schulz C, Ahlers O, Bercker S, Volk HD, Doecke WD, Falke KJ, Gerlach H (2003). Immunologic and hemodynamic effects of “low-dose” hydrocortisone in septic shock: a double-blind, randomized, placebo-controlled, crossover study. Am J Respir Crit Care Med.

[41] Marik PE (2018). Steroids for sepsis: yes, no or maybe. J Thorac Dis.

[42] Lee N, Allen Chan KC, Hui DS, Ng EK, Wu A, Chiu RW, Wong VW, Chan PK, Wong KT, Wong E (2004). Effects of early corticosteroid treatment on plasma SARS-associated coronavirus RNA concentrations in adult patients. J Clin Virol.

[43] Ni YN, Chen G, Sun J, Liang BM, Liang ZA (2019). The effect of corticosteroids on mortality of patients with influenza pneumonia: a systematic review and meta-analysis. Crit Care.

[44] Venkatesh B, Finfer S, Cohen J, Rajbhandari D, Arabi Y, Bellomo R, Billot L, Correa M, Glass P, Harward M (2018). Adjunctive glucocorticoid therapy in patients with septic shock. N Engl J Med.

[45] Villar J, Ferrando C, Martínez D, Ambrós A, Muñoz T, Soler JA, Aguilar G, Alba F, González-Higueras E, Conesa LA (2020). Dexamethasone treatment for the acute respiratory distress syndrome: a multicentre, randomised controlled trial. Lancet Respir Med.

[46] Russell CD, Millar JE, Baillie JK (2020). Clinical evidence does not support corticosteroid treatment for 2019-nCoV lung injury. Lancet.

[47] Shang L, Zhao J, Hu Y, Du R, Cao B (2020). On the use of corticosteroids for 2019-nCoV pneumonia. Lancet.

[48] Langarizadeh MA, Ranjbar Tavakoli M, Abiri A, Ghasempour A, Rezaei M, Ameri A (2021). A review on function and side effects of systemic corticosteroids used in high-grade COVID-19 to prevent cytokine storms. EXCLI J.

[49] Nicolau DV, Bafadhel M (2020). Inhaled corticosteroids in virus pandemics: a treatment for COVID-19?. Lancet Respir Med.

[50] Taiwan Centers for Disease Control: Treatment and criteria of release from quarantine for confirmed COVID-19 patient, 2021/05/17 version. 2021, Available at https://www.cdc.gov.tw/Uploads/ccc5db1d-8234-4235-a91e-f4547eff76c5.pdf?fbclid=IwAR07x5kmq5RJ-VUv8LNd7n43OfCgXF_NKsrrljsBdU33M1hBbuRglmMl6Kc; Accessed 12 Feb 2022

[51] Lee S, Kim T, Lee E, Lee C, Kim H, Rhee H, Park SY, Son HJ, Yu S, Park JW (2020). Clinical course and molecular viral shedding among asymptomatic and symptomatic patients with SARS-CoV-2 infection in a community treatment center in the Republic of Korea. JAMA Intern Med.

[52] Taiwan Centers for Disease Control: Cumulative number of vaccinations for various types of COVID-19 vaccine (until 2021/06/21). 2021, Available at https://www.cdc.gov.tw/En/File/Get/GKFehCWe5feNtzpATZfhlg. Accessed 09 Mar 2022.

[53] Malik P, Patel U, Mehta D, Patel N, Kelkar R, Akrmah M, Gabrilove JL, Sacks H (2021). Biomarkers and outcomes of COVID-19 hospitalisations: systematic review and meta-analysis. BMJ Evid-Based Med.

[54] Patanavanich R, Glantz SA (2021). Smoking is associated with worse outcomes of COVID-19 particularly among younger adults: a systematic review and meta-analysis. BMC Public Health.

