# Supplementary material for: Clinical benefits of inhaled ciclesonide for hospitalized patients with COVID-19 infection: a retrospective study
Source: BMC Pulm Med. 2022 Sep 28;22:368. doi: 10.1186/s12890-022-02168-8 (PMC9517967; doi:10.1186/s12890-022-02168-8)
Supplement: Supplementary file 1 — Additional file 1: 1 Treatment protocol for COVID patients at MacKay Memorial Hospital (2021). 2 Admission order set (2021). [file 12890_2022_2168_MOESM1_ESM.docx]

**Supplement 1. Treatment protocol for OVID patients at MacKay Memorial Hospital (2021)**


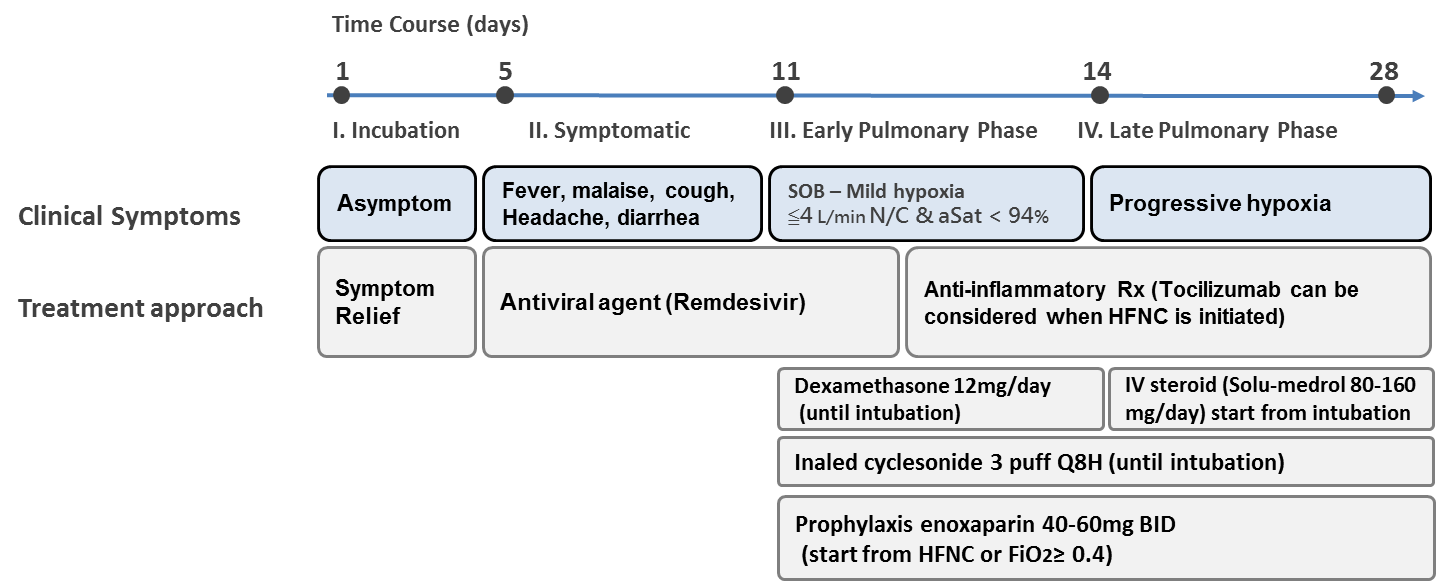


**Supplement 2. Admission order set (2021)**

On the service of Dr. X

Diagnosis: COVID-19 infection

Allergy: medication/food

Nursing care: Level B/C personal protective equipment

◆◆◆ ◆◆◆ Please write the severity score on the admission note ◆◆◆ ◆◆◆

Severity system for predicting mortality for severe patients with COVID-19 (Y. Shang et al. 2020)

Age 60-75 y/o +1

Age 75 y/o +2

Coronary heart disease +1

Lymphocyte < 8% +1

Procalcitonin > 0.15 ng/ml +2

D-dimer > 500 ng/mL +1

High risk: Total score > 2 points

Low risk: Total score ≤ 2 points

◆◆◆◆◆◆◆◆◆◆◆◆◆◆◆◆◆◆◆◆

**Isolation Care Principles:**

1. Make a work plan before entering the isolation area to reduce unnecessary entry and exit; the time limit is less than 30 minutes.

2. Blood test items to be detected: CBC/DC, PT/aPTT, BUN/Cr, GOT/GPT, total bilirubin, CRP, LDH, d-dimer, ferritin, procalcitonin; if the patient has a fever, collect two blood culture sets.

3. Portable CXR.

4. Monitor SpO2. If room air SpO2 < 94%, follow-up arterial blood gas, portable CXR, and CRP.

5. If the patient’s CXR shows pneumonia, follow-up with portable CXR the next day.

6. Medication treatment: bromhexine 8 mg TID, + symptomatic treatment.

7. Consult a traditional Chinese medical doctor for traditional Chinese medicine NRICM101 if the patient requests it.

8. If the patient has hypoxia with oxygen use, prescribe inhaled ciclesonide 3PF Q8H, dexamethasone 6 mg IV or PO, consider using remdesivir.

9. If FiO2 is > 0.4, contact the Department of Anesthesiology for possible intubation.

10. For those intubated with mechanical ventilation, prescribe anti-IL6 medication (tocilizumab).

11. Perform a second COVID RT-PCR if the patient’s fever subsides for at least 1 day and 10 days after the onset of the disease (asymptomatic cases are calculated on the day of collection).
